# AI as a Medical Device for Ophthalmic Imaging in Europe, Australia, and the United States: Protocol for a Systematic Scoping Review of Regulated Devices

**DOI:** 10.2196/52602

**Published:** 2024-03-14

**Authors:** Ariel Yuhan Ong, Henry David Jeffry Hogg, Aditya U Kale, Priyal Taribagil, Ashley Kras, Eliot Dow, Trystan Macdonald, Xiaoxuan Liu, Pearse A Keane, Alastair K Denniston

**Affiliations:** 1 Moorfields Eye Hospital NHS Foundation Trust London United Kingdom; 2 Oxford Eye Hospital Oxford University Hospitals NHS Foundation Trust Oxford United Kingdom; 3 Population Health Sciences Institute Faculty of Medical Sciences Newcastle University Newcastle United Kingdom; 4 Department of Ophthalmology Queen Elizabeth Hospital Birmingham University Hospitals Birmingham NHS Foundation Trust Birmingham United Kingdom; 5 Institute of Inflammation and Ageing University of Birmingham Birmingham United Kingdom; 6 Sydney Eye Hospital Sydney Australia; 7 Retinal Consultants Medical Group Sacramento, CA United States; 8 NIHR Birmingham Biomedical Research Centre Birmingham United Kingdom; 9 Centre for Regulatory Science and Innovation Birmingham Health Partners Birmingham United Kingdom; 10 Institute of Ophthalmology University College London London United Kingdom; 11 NIHR Moorfields Biomedical Research Centre London United Kingdom

**Keywords:** AIaMD, artificial intelligence as a medical device, artificial intelligence, deep learning, machine learning, ophthalmic imaging, regulatory approval

## Abstract

**Background:**

Artificial intelligence as a medical device (AIaMD) has the potential to transform many aspects of ophthalmic care, such as improving accuracy and speed of diagnosis, addressing capacity issues in high-volume areas such as screening, and detecting novel biomarkers of systemic disease in the eye (oculomics). In order to ensure that such tools are safe for the target population and achieve their intended purpose, it is important that these AIaMD have adequate clinical evaluation to support any regulatory decision. Currently, the evidential requirements for regulatory approval are less clear for AIaMD compared to more established interventions such as drugs or medical devices. There is therefore value in understanding the level of evidence that underpins AIaMD currently on the market, as a step toward identifying what the best practices might be in this area. In this systematic scoping review, we will focus on AIaMD that contributes to clinical decision-making (relating to screening, diagnosis, prognosis, and treatment) in the context of ophthalmic imaging.

**Objective:**

This study aims to identify regulator-approved AIaMD for ophthalmic imaging in Europe, Australia, and the United States; report the characteristics of these devices and their regulatory approvals; and report the available evidence underpinning these AIaMD.

**Methods:**

The Food and Drug Administration (United States), the Australian Register of Therapeutic Goods (Australia), the Medicines and Healthcare products Regulatory Agency (United Kingdom), and the European Database on Medical Devices (European Union) regulatory databases will be searched for ophthalmic imaging AIaMD through a snowballing approach. PubMed and clinical trial registries will be systematically searched, and manufacturers will be directly contacted for studies investigating the effectiveness of eligible AIaMD. Preliminary regulatory database searches, evidence searches, screening, data extraction, and methodological quality assessment will be undertaken by 2 independent review authors and arbitrated by a third at each stage of the process.

**Results:**

Preliminary searches were conducted in February 2023. Data extraction, data synthesis, and assessment of methodological quality commenced in October 2023. The review is on track to be completed and submitted for peer review by April 2024.

**Conclusions:**

This systematic review will provide greater clarity on ophthalmic imaging AIaMD that have achieved regulatory approval as well as the evidence that underpins them. This should help adopters understand the range of tools available and whether they can be safely incorporated into their clinical workflow, and it should also support developers in navigating regulatory approval more efficiently.

**International Registered Report Identifier (IRRID):**

DERR1-10.2196/52602

## Introduction

### Overview

There is a growing capacity-demand mismatch within ophthalmology, increasing the risk of sight loss from treatment delays [1,2]. Artificial intelligence (AI) has the potential to help address these challenges. AI’s strength lies in its ability to produce high-throughput analyses and glean meaningful insights from complex multimodal and multidimensional data sets through pattern recognition. This is well aligned with ophthalmology services, where disease diagnosis and management depend heavily on multimodal imaging [3]. AI therefore has the potential to help improve speed and access to care at reduced costs.

However, despite the exponential increase in the number of AI as a medical device (AIaMD) being developed and receiving regulatory approval, relatively few have been seamlessly integrated into routine clinical practice [4-6]. This so-called “AI chasm” limits the deployment of AIaMD to achieve patient benefit at scale [7]. This AI chasm results from a wide range of interdependent factors at the policy, organizational, and individual levels [8-13]. Key elements include ensuring adequate clinical evaluation to support regulatory decisions, such that the evidence base underpinning such tools is aligned with their intended use and is safe for the target population, and also clarifying how these regulatory requirements align with commissioners’ needs.

The evidential requirements for software (including AIaMD) may be more ambiguous compared to more established interventions, making it more difficult for AI developers to explicitly understand the nature and extent of evidence they need to generate to gain regulatory approval. Attempts to study regulator approved AIaMD are impeded by the usability of public databases as well as the private nature of much of the information submitted by applicants, making it difficult for researchers, clinicians, or commissioners attempting to understand the evidence underpinning approved AIaMD. A review of Food and Drug Administration (FDA) approvals in the United States found that few submissions included comparisons between AI and human performance and that only a small proportion reported prospective data [14]. The reporting of sample size and number of sites in the validation studies was generally poor. The review did not assess whether participant characteristics such as gender and ethnicity were reported. Although guidelines for performing and presenting AI studies have been developed [15-18], there is no clear “best practice” for providers to ensure the safety and effectiveness of the AIaMD they adopt [14].

### Review Objectives

This scoping review will focus on AIaMD that contributes to clinical decision-making (relating to screening, diagnosis, prognosis, and treatment) using ophthalmic imaging as an input. The aim is to identify and characterize AIaMD for ophthalmic imaging, which have received regulatory approval in 4 countries with established regulatory pathways for clinical use, in order to support providers in procurement decisions and developers in generating evidence to support applications to regulators.

The objectives are as follows:

1. To identify regulator-approved AIaMD for ophthalmic imaging in Europe, Australia, and the United States
2. To report the characteristics of those AIaMD and the regulatory approvals granted to them
3. To report the available evidence for the effectiveness and efficacy of approved AIaMD

## Methods

### Protocol Registration and Reporting

The protocol and subsequent review will adhere to the PRISMA-P (Preferred Reporting Items for Systematic Review and Meta-Analysis Protocols) [19] and PRISMA-ScR (Preferred Reporting Items for Systematic Reviews and Meta-Analyses extension for Scoping Reviews) [20] reporting guidelines, respectively (note that PRISMA-AI [21] is still in development). The protocol was registered with the Open Science Framework’s website.

### Eligibility Criteria

This review will focus on AIaMD for ophthalmic imaging that helps inform clinical management. All AIaMD for ophthalmic imaging where the AIaMD or its manufacturer are recorded in the US FDA, Medicines and Healthcare products Regulatory Agency (MHRA), European Database on Medical Devices (EUDAMED), or Australian Register of Therapeutic Goods (ARTG) databases will be included. All 4 countries are members of the International Medical Regulators Device Forum and have a track record of admitting AIaMD to their markets. No restrictions will be placed on the type of ophthalmic imaging modality involved or the intended use of the AIaMD.

The AIaMD will have a partial or fully data-led mechanism (eg, regression modeling, random forest, or convolutional neural networks). Any AIaMD that exclusively uses rule-based mechanisms (eg, a priori decision trees, best practice alerts, and normal or abnormal threshold alerts) will be excluded.

With regard to the evidence underpinning each AIaMD, only original research that comprises a clinical evaluation of the AIaMD in human participants will be included. This may include randomized controlled trials or prospective or retrospective observational studies. Systematic reviews and meta-analyses, case series, case reports, commentaries, and expert opinions will not be eligible. No date or language restrictions will be applied to the electronic search.

### Search Strategy and Sources of Information

To identify potentially eligible AIaMD, the FDA (United States), ARTG (Australia), MHRA (United Kingdom), and EUDAMED (European Union) regulatory databases will be searched through a snowballing approach [22]. This will involve an exhaustive review of the product class codes and predicate devices (if applicable) with which each known eligible device is associated. This strategy has been adopted due to limitations in the search functionality of these databases. No AI tools will be used to assist the search.

The snowball search will commence with a list of 15 AIaMD for ophthalmic imaging (Textbox 1). This represents the sum of the authors’ awareness of regulated products and a pragmatic search of relevant academic literature [23].

**Initial list of ophthalmic imaging artificial intelligence as a medical device (AIaMD) used in snowball search.**

**Ophthalmic imaging AIaMD**

- LumineticsCore (previously known as IDx-DR), Digital Diagnostics
- Eyeart, Eyenuk Inc
- RetmarkerDR, Retmarker SA
- SELENA+, eyRIS Pte Ltd
- Automated Retinal Disease Assessment, Verily Life Sciences
- Medios AI, Remidio
- OphtAI, Evolucare, ACDIS
- RetCAD, Thirona Retina B.V.
- DeepDee AI, DeepDee
- MONA DR, MONA
- Eyetelligence, Eyetelligence Pty Ltd
- CARA, Diagnos
- RetinaLyze, RetinaLyze System A/S (Ltd)

Next, PubMed, ClinicalTrials.gov, and the International Clinical Trials Registry Platform will be systematically searched for each eligible AIaMD and its manufacturer by combining both search terms with an “OR” Boolean operator. These searches will be limited to relevant ophthalmology-specific studies using relevant key terms such as “retin*” for AIaMD relating to diabetic retinopathy screening.

In addition, manufacturers’ websites will also be reviewed for any peer-reviewed publications. The manufacturers of all eligible AIaMD will be contacted directly for clarification and as an additional source of peer-reviewed publications and ongoing studies. A preliminary scoping search highlighted that not all studies mention the AI device name or manufacturer, and some devices undergo a name change from one version to the next. Hence, this additional search will ensure that the data captured are as comprehensive as possible.

### Study Selection

Two authors will search the regulatory databases independently. They will come to a consensus decision about the eligibility of any AIaMD identified. Any unresolved disagreements will be arbitrated by a third author. There may be instances where an AIaMD’s eligibility or its regulatory approval status cannot be determined with publicly available evidence, as eligible devices may use proprietary AI that is kept confidential. If this is the case, correspondence with the manufacturer will be undertaken to seek clarification. If further clarification is not possible, the ambiguity about the AIaMD’s eligibility and the rationale for including or excluding it will be recorded.

The search for evidence will be undertaken by an independent author. After deduplication, the titles and abstracts will be independently screened by 2 authors to assess their relevance to the eligible AIaMD. Discrepancies will be resolved by discussion and by arbitration with an additional author if necessary. The full texts will be screened, and a further round of arbitration will take place as needed.

### Data Extraction

Data extraction will be undertaken in 2 phases, using standardized data extraction forms designed and piloted for the purposes of this review.

#### Phase 1

The characteristics of each eligible AIaMD for ophthalmic imaging with regulatory approval in Europe, Australia, and the United States at the time of the search will be obtained. Where available, this will include:

1. Type of regulator approval and date of approval
2. Class assigned under FDA, TGA, UK MDR (Medical Devices Regulations 2002) and/or EU MDR (Regulation (EU) 2017/745)
3. Intended use statement
4. Ophthalmic imaging modality
5. Model type and architecture (eg, deep learning with machine learning)
6. Recall indications on the regulatory databases, as available

Some AIaMD may be approved in 2 or more jurisdictions, and the data will be extracted accordingly. Google searches will be used to supplement data extraction if the required data are unavailable from the FDA, EUDAMED, MHRA, or ARTG databases.

#### Phase 2

Published evidence underpinning the effectiveness and efficacy of each eligible AIaMD will be obtained. The following data will be extracted from each included study:

1. Study characteristics: title, author name, publication status, funding source, conflicts of interest, and author affiliations with manufacturers
2. Study methodology: study duration, study design (randomized, prospective observational, and retrospective observational), etc
3. Validation: external validation, reference standards, and comparison between AI and humans
4. Data set or cohort details: source of data set, size of data set or number of participants, setting, number of countries, number of centers, and participant demographics (age, gender, and ethnicity)
5. Model performance: metrics including sensitivity, specificity, area under the curve, etc, with 95% CIs; clinical outcomes as described

### Assessment of Methodological Quality

Two review authors will independently assess the methodological quality of each included clinical validation study. Appropriate quality assessment tools will be used for each study type, for example, the QUADAS-2 tool for evaluating the risk of bias and applicability of primary diagnostic accuracy studies [24], the Cochrane risk of bias-2 tool for randomized controlled trials [25], and the ROBINS-I (Risk of Bias in Nonrandomized Studies of Interventions) tool for nonrandomized studies [26]. QUADAS-AI (quality assessment tool for artificial intelligence-centered diagnostic test accuracy studies) [27] and PROBAST-AI (Prediction Model Risk of Bias Assessment Tool for Artificial Intelligence) [28] are under development at the time of writing but will be used where appropriate if available. Any disagreements will be resolved through discussion or by involving a third reviewer where consensus cannot be reached.

### Data Synthesis

#### Study-Level Data

The extracted data will be synthesized using narrative and tabular approaches. A summary of the findings will be presented, using descriptive statistics to describe the characteristics of the included studies. For example, mean and SD will be used to describe continuous variables, while percentages will be used to describe proportions. If appropriate, a meta-analysis of AIaMD diagnostic accuracy will be considered, but this may not be feasible if there is significant heterogeneity in study methods and AI methodology.

#### AIaMD-Level Data

The data for each AIaMD will be synthesized to give an overview of the characteristics of its regulatory approval or approvals and its clinical validation studies, again through narrative and tabular approaches.

### Ethical Considerations

Ethical approval is not required, as this is a protocol for a systematic scoping review. All relevant data have been published, and no primary or proprietary data will be collected. This decision has been verified with the Newcastle University Ethics Committee.

## Results

Preliminary searches were conducted in February 2023, and screening is underway. Data extraction, data synthesis, and assessment of methodological quality commenced in October 2023. We anticipate that the scoping review will be completed and submitted for peer review by April 2024.

## Discussion

This will be the first review to examine and synthesize evidence on AIaMD for ophthalmic imaging. The key aim is to better understand the landscape of AIaMD for ophthalmic imaging on the market and the level of evidence that supports their regulatory approval.

### Strengths and Limitations

Due to the limited and variable functionality of regulatory databases, it was not possible to conduct searches with standard systematic review methodologies based on research databases. This has necessitated several design considerations. We have sought to minimize publication bias and improve the completeness of the search process by undertaking a snowballing approach informed by expert knowledge, in combination with a database search. This was necessitated by limitations in the search functionality of the regulatory databases, but we acknowledge that this approach facilitates the mitigation rather than the removal of these limitations. In addition, our PubMed search strategy may be hampered by incomplete reporting or no mention of the AI device’s name or manufacturer. We have attempted to mitigate this by supplementing our search with reviewing manufacturers’ websites, corresponding with manufacturers, and performing adjunct searches of major clinical trial registries in order to ensure that our search is as comprehensive as possible.

Strengths include the international scope of the review. We recognize that there may be differences in regulatory requirements across territories (and in the transparency of reporting of data supporting those applications) and have therefore included 4 major jurisdictions. We have also assembled an international team of authors with representation from the United States, Europe, and Australia to support the interpretation of the data. The inclusion of critical appraisal to identify any methodological variations or shortcomings of existing clinical validation studies is a further strength not common to previous regulatory reviews of AIaMDs, and will help guide any future improvements.

### Conclusion

We describe the protocol for a systematic scoping review that seeks to map and examine AIaMD for ophthalmic imaging that has received regulatory approvals for commercial use. We anticipate that our findings may be of interest to ophthalmic professionals, AI model developers, health care commissioners, and policy makers, with the overall aim of improving transparency to help inform safe AIaMD implementation, thereby optimizing patient care.

## Abbreviations

AI: artificial intelligence
AIaMD: artificial intelligence as a medical device
ARTG: Australian Register of Therapeutic Goods
EUDAMED: European Database on Medical Devices
FDA: Food and Drug Administration
MHRA: Medicines and Healthcare products Regulatory Agency
PRISMA-P: Preferred Reporting Items for Systematic Review and Meta-Analysis Protocols
PRISMA-ScR: Preferred Reporting Items for Systematic Reviews and Meta-Analyses extension for Scoping Reviews
PROBAST-AI: Prediction Model Risk of Bias Assessment Tool for Artificial Intelligence
QUADAS-AI: quality assessment tool for artificial intelligence–centered diagnostic test accuracy studies
ROBINS-I: Risk of Bias in Nonrandomized Studies of Interventions

## Data Availability

Data sharing is not applicable to this article, which is a protocol for a scoping review, as no data sets were generated or analyzed.
